# Retrospective Analysis of Pediatric Foreign Body Ingestion: A Decade of Emergency and Specialty Care at King Saud University Medical City (2013–2023)

**DOI:** 10.1155/emmi/4939829

**Published:** 2026-08-25

**Authors:** Hani A. Alghamdi, Maram A. Aldeej, Rola K. Alzahrani, Reema A. Alrashidi, Tarfa N. Albaz, Sadeem A. Alhazmi, Lama A. Al-Eyadhy, Shouq A. Alhathal, Ghadah H. Alqahtani

**Affiliations:** ^1^ Department of Family and Community Medicine, College of Medicine, King Saud University, Riyadh, Saudi Arabia, ksu.edu.sa; ^2^ College of Medicine, King Saud University, Riyadh, Saudi Arabia, ksu.edu.sa

**Keywords:** button battery ingestion, coin ingestion, emergency medicine, esophageal foreign body, pediatric emergency, pediatric foreign body ingestion

## Abstract

**Background:**

Foreign body ingestion (FBI) is a common pediatric emergency with variable clinical outcomes. Following the introduction of coin currency in Saudi Arabia in 2017, a notable shift in ingestion patterns has been reported. This study aimed to evaluate the epidemiology, clinical characteristics, management strategies, and outcomes of pediatric FBI cases presenting to King Saud University Medical City (KSUMC), Riyadh, over a 10‐year period.

**Methods:**

A retrospective cohort study was conducted including children aged ≤ 14 years who presented with FBI between 2013 and 2023. Cases were identified through electronic medical records. Data collected included demographics, clinical presentation, type and site of foreign body, diagnostic modality, management approach, complications, admission status, and length of stay (LOS). Comparative analyses were performed across time periods, and regression models were used to identify factors associated with hospital admission and LOS. Statistical significance was set at *p* < 0.05.

**Results:**

A total of 89 pediatric patients were included, with a median age of 5 years (IQR 3, 8); 68.5% were male. Coins were the most commonly ingested objects (58.4%), followed by button batteries (11.2%). The esophagus was the most frequent site of impaction (48.3%) and was strongly associated with endoscopic intervention (*p* < 0.001), whereas gastric and distal bowel foreign bodies were predominantly managed conservatively. Symptomatic presentation significantly increased the likelihood of hospital admission, particularly in patients presenting with vomiting or drooling. Increasing age independently predicted both admission and longer LOS, while observation‐only management was associated with reduced admission rates and shorter hospitalization. Complications were uncommon (4.5%), with no major adverse outcomes observed.

**Conclusion:**

Coin ingestion remains the predominant form of pediatric FBI following Saudi Arabia’s currency transition, with esophageal impaction driving the need for endoscopic intervention. Age, symptomatology, and site of impaction significantly influence admission and LOS. These findings support risk‐stratified triage, conservative management for low‐risk cases, and targeted preventive strategies, particularly for high‐risk objects, such as button batteries. A risk‐stratified emergency management framework, summarized in a decision‐making flowchart, supports safe triage of pediatric FBIs in tertiary emergency settings.

## 1. Introduction

Ingestion of foreign bodies (FBs) is a common pediatric problem, with more than 100,000 cases occurring each year in the United States [1]. The types of ingested FBs differ among countries according to feeding habits, cultural features, and sociocultural properties [2]. Notably, metallic coins represent the predominant inorganic form of ingested FBs, constituting approximately 53% of such incidents in the pediatric population [3]. Ingestion and subsequent esophageal retention of coins can precipitate severe clinical manifestations and complications. Untreated cases may progress to intestinal obstruction, perforation, hemorrhage, abscess development, and respiratory compromise [4, 5]. Endoscopic intervention remains the standard and most widely accepted method for the retrieval of coins lodged in the esophagus [5]. Additionally, swallowing button batteries can be life‐threatening, especially if they block the esophagus. Global efforts are in place to prevent, detect, and treat cases of button battery ingestion [6]. Notably, in December 2017, Saudi Arabia introduced 1‐riyal and 2‐riyal coins to replace the existing 1‐riyal banknote [5].

FBs encountered in clinical practice can be broadly classified into three categories based on their physical characteristics: first, true FBs, including smooth objects, such as coins, toys, and marbles; second, sharp or pointed objects, such as pins, needles, toothpicks, and bone fragments; and third, large food boluses that may cause esophageal impaction. This classification is of direct clinical relevance because the shape and composition of the ingested object are primary determinants of urgency. For instance, smooth blunt objects (e.g., coins) in the stomach or distal bowel without symptoms may be managed conservatively with clinical and radiographic follow‐up, with elective endoscopic removal considered if they fail to progress or pass [7]. In contrast, sharp or pointed foreign bodies carry a higher risk of gastrointestinal perforation and generally warrant prompt endoscopic assessment and removal when accessible although management may vary according to their anatomical location, symptoms, and evidence of progression [7]. Similarly, button batteries impacted in the esophagus represent a distinct emergency category necessitating removal, preferably within 2 hours of presentation, due to their ability to generate hydroxide ions through an electrochemical reaction causing rapid alkaline injury and potentially fatal liquefactive necrosis [6, 7].

Children have an increased tendency to explore and swallow different types of objects, such as coins, buttons, batteries, magnets, or toys [8]. FB ingestion was higher in children whose age was between 6 months and 6 years old, owing to their exploratory habits [9]. The widespread use of button batteries in electronic devices has been associated with an increasing incidence of accidental ingestion [10], as poorly secured devices contributed to the problem [6]. Since the implementation of coin currency in 2017, the incidence of FB ingestion has increased more than five times [5]. This increase is relatively related to the increase in coins as a FB. A regional study carried out in December 2022 documented 104 cases of foreign body ingestion (FBI) over 32 months; before the currency change, coins accounted for merely 2 cases (10%), which surged to 83 cases (79.8%) following the introduction of new coinage [5].

Anatomically, the location of FB impaction is commonly in the narrowest parts, such as the esophagus and pyloric sphincter [6, 8]. However, a regional study found that the stomach is the most common/likely site of impaction [11].

The diagnostic approach varies by the suspected type and composition of the ingested object. Plain radiography of the neck, chest, and abdomen is the recommended first‐line investigation and reliably identifies radiopaque objects, such as coins, metallic sharp objects, and button batteries [7]. However, radiolucent objects including fish and chicken bones, plastic items, wooden fragments, and glass are frequently invisible on standard plain films [12]. In such cases, computed tomography (CT) offers superior sensitivity and is particularly valuable when esophageal perforation or deep tissue involvement is suspected [13]. Nevertheless, a proportion of ingested FBs cannot be confirmed by any noninvasive imaging modality, regardless of the technique employed. In these patients, upper gastrointestinal endoscopy serves simultaneously as the definitive diagnostic and therapeutic tool, and clinicians must therefore maintain a high index of suspicion based on clinical history and witnessed ingestion even in the presence of unremarkable imaging findings [7, 14].

Generally, the primary management of FBI was spontaneous passage. Reassurance and clinical treatment with fecal observations or follow‐up radiography after a few days were advised. Endoscopic removal, including esophagoscopy, gastroscopy, and esophagogastroduodenoscopy, depends on the type of the ingested FB, the button battery, delayed seeking hospital (> 24 h), the duration of the stay, and surgical or endoscopic intervention [11, 15]. The most commonly used procedure for the removal of FB was upper endoscopy. However, the population of this study included those treated either by endoscopy or laparotomy [8].

Complications are defined as any incident involving significant morbidity, such as esophageal stricture, esophageal perforation, and esophageal fistula [5, 15]. Aspiration pneumonia, intestinal perforation, enteric fistula formation, and peritonitis were defined as the major FB ingestion complications [5, 15]. A local injury at the location of FB impaction, such as an ulcer or erosion, was considered a minor complication [11]. There was a significant relationship between the type of FB and complications, with 1% of coin FB and 19% of battery FB ingestion cases needing to stay in the hospital for more than 24 h [10]. There was a statistically significant relationship between the types of FB and the need for rigid upper endoscopy [10]. Regarding esophagoscopy findings, the percentage of coin ingestion cases linked to complications was 1%, whereas 71.4% of battery ingestion cases had complications [10].

Different studies over the years in various places have indicated variations in the prevalence and incidence of FB ingestion among pediatric patients due to the diversity of FB types and the anatomical location of FB. This study extends the work of previously conducted research at KKUH. However, the time frame of the Wafi et al. study spanned from 1995 to 2013, which was before the implementation of coin currency in Saudi Arabia, which took place in December of 2017 [5]. Wafi et al. subsequently demonstrated a marked increase in coin ingestion incidence [5]. Therefore, this study aimed to characterize the prevalence of FB ingestion and analyze the epidemiology, clinical presentations, findings, and outcomes of a cohort of pediatric patients who presented to King Saud University Medical City (KSUMC), Riyadh, Saudi Arabia, between 2013 and 2023. Note: KSUMC comprises King Khalid University Hospital (KKUH) and King Abdulaziz University Hospital (KAUH), both of which contributed cases to this analysis. Additionally, it aims to improve local statistics on various types of FB ingestion, management, and outcomes, as well as raise awareness of the risks associated with FB ingestion and encourage parents to keep such products out of children’s reach. This can be done by implementing preventive measures, new policies, and teaching campaigns in nurseries, schools, and on social media.

## 2. Materials and Methods

A retrospective cohort study was conducted. All medical records of pediatric patients aged 14 years or younger who presented with FBI and were managed at the emergency department or pediatric services at KSUMC, Riyadh, Saudi Arabia, between 2013 and 2023 were retrospectively reviewed. Patients were identified through the electronic medical record system based on diagnostic coding for FBI. Ingestion was classified as “intentional” only when the medical record explicitly documented a caregiver or healthcare provider’s description of the event as deliberate, typically in older children (> 8 years of age). In younger children, all ingestion events were classified as accidental as reported. This classification was based exclusively on documented caregiver history and was not independently verified. Of 154 patient records identified through the electronic medical record system using diagnostic codes for FBI, 19 were excluded for not meeting the age criterion of ≤ 14 years, yielding 135 eligible pediatric patients. A further 46 were excluded due to incomplete documentation across one or more key study variables, including diagnostic approach, FB type, anatomical site of impaction, intervention category, and presentation characteristics, resulting in a final analytic cohort of 89 patients.

**FIGURE 1:**
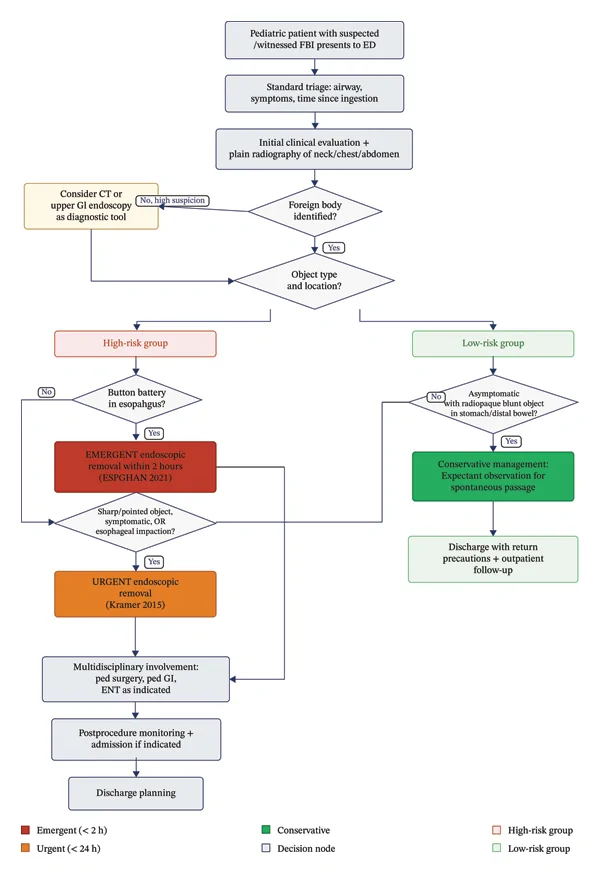
Risk‐stratified emergency management pathway for pediatric foreign body ingestion (institutional framework) ^∗^ [6, 7]. ^∗^This flowchart represents the general institutional management framework and is not derived directly from retrospective patient‐level data.

Demographic information, clinical presentation, type of FB ingested, diagnostic modality, management approach, need for endoscopic or surgical intervention, hospital course, complications, and clinical outcomes were collected. FBI was defined as documented ingestion of a nonfood object confirmed clinically, radiographically, and/or endoscopically. Diagnostic evaluation was performed according to institutional protocols and primarily involved plain radiography, with CT utilized in selected cases when clinically indicated. Management strategies were categorized based solely on what was documented in the medical records, into three groups: (i) endoscopic removal, which included upper gastrointestinal endoscopy performed with therapeutic intent; (ii) conservative management, defined as expectant observation with anticipation of spontaneous passage, applied predominantly to asymptomatic patients with FBs located in the stomach or distal bowel; and (iii) other procedures, which encompassed direct laryngoscopy and surgical endoscopy for FBs at sites not amenable to conventional upper endoscopy. At KSUMC, initial emergency department assessment includes evaluation of airway patency, symptomatology, estimated time since ingestion, and identification of the ingested object type when possible; plain radiography of the neck, chest, and abdomen is the routine first‐line imaging investigation for suspected radiopaque FBs, with CT reserved for selected cases in which the diagnosis cannot be established radiographically or in which perforation is suspected. In line with international guidance [6, 7], the institutional framework prioritizes risk stratification based on FB type, anatomical location, and symptom status: High‐risk presentations (esophageal impaction, symptomatic patients, button batteries, sharp or pointed objects, and cases with suspected complications) are managed with urgent or emergent endoscopic intervention, whereas lower risk presentations (asymptomatic patients with radiopaque blunt objects lodged in the stomach or distal bowel) are typically managed conservatively with expectant observation for spontaneous passage. The general institutional decision‐making pathway is summarized in Figure 1; because urgency categories and detailed decision rationales were not consistently documented at the patient level in the retrospective records, individual patients have not been re‐classified against this framework post hoc. Detailed procedural variables including the specific type of endoscope, retrieval instrumentation, anesthesia protocol, and technical approach were not systematically documented in the electronic medical records and are therefore not reported here; this constraint is acknowledged in the Limitations section. Mortality and recurrence or repeat ingestion events were not assessed as primary outcomes in this study. In‐hospital mortality was not documented in any patient during the study period; however, postdischarge mortality and out‐of‐hospital events were outside the scope of electronic medical record review. Similarly, recurrent ingestion events presenting to other facilities could not be captured through our single‐center record system. These outcomes are therefore reported as absent from the current analysis rather than as confirmed zero‐event findings and should be prospectively assessed in future studies. Emergency‐specific outcomes, such as complication timing relative to ingestion, granular emergency department length of stay (LOS) stratified by triage acuity, and pediatric intensive care unit admission, were not consistently captured in the medical records and are therefore not reported.

Statistical analysis was performed using R statistical software. Statistical analyses were divided into descriptive and inferential components. Descriptive statistics were used to summarize patient demographics and characteristics of the ingested FBs. Measures of central tendency, including means and medians, were used to describe age and duration of hospital stay, while variability was expressed using standard deviations and interquartile ranges. Frequencies and percentages were used to categorize the types of ingested FBs and calculate incidence rates. For inferential analysis, chi‐square tests were employed to assess differences in categorical variables, including comparisons of FBI patterns before and after the introduction of new coin denominations. Logistic regression analysis was performed to evaluate the relationship between patient characteristics, type of FB ingested, and the likelihood of developing complications. Time series analysis was conducted to determine trends in FBI incidence over the study period. These inferential methods were used to identify associations and potential contributory factors related to risk and prevention. Given the modest sample size (*n* = 89) relative to the number of covariates entered into the multivariable models, regression results are interpreted as exploratory and hypothesis‐generating rather than confirmatory; the potential for overfitting and the wide confidence intervals observed in several adjusted estimates are further discussed in the Limitations. Hypothesis testing was performed at a 5% level of significance.

This study was approved by the Institutional Review Board of the College of Medicine at King Saud University (IRB Approval Number: E‐23‐8328). A waiver of informed consent was granted by the Institutional Review Board given the retrospective, noninterventional design and the use of de‐identified data extracted from routinely collected medical records. Obtaining individual informed consent from 89 patients or their guardians over a 10‐year period was deemed impracticable given the extended study window, the pediatric population, and the anticipated loss to contact for a substantial proportion of families. The study posed no more than minimal risk to participants, no patient‐identifying information was retained in the analytic dataset, and all data handling complied with institutional data‐protection standards. The study was conducted in accordance with the ethical principles of the Declaration of Helsinki.

## 3. Results

A total of 89 patients (61 males and 28 females) with a median age of 5 years (IQR 3, 8) were included in this analysis, the majority of whom (91%) were Saudi. Most patients (87.6%) had no medical conditions, while 12.4% had medical conditions, such as asthma. Moreover, 53.9% of patients were reported at KKUH and the remainder at KAUH. Presentation of cases was unwitnessed in 32.6% and witnessed in 67.4%. Most events (96.6%) were accidental, while 3.4% were intentional. Furthermore, 69.7% of patients were symptomatic, and the median time to ER admission was 120 (IQR 60, 360) hours (Table 1). Applying the institutional risk‐stratification framework, the cohort included both higher risk presentations (esophageal impaction, *n* = 43 [48.3%]; symptomatic presentation, *n* = 62 [69.7%]; button battery ingestion, *n* = 10 [11.2%]) and lower risk presentations (asymptomatic gastric or distal bowel FBs). This risk‐stratified distribution informed the management pathway summarized in Figure 1.

**TABLE 1:** Baseline characteristics of the studied patients (*n* = 89).

Item	*N*	%
*Age (years)*		
Median (IQR)	5 (3, 8)	

*Gender*		
Male	61	68.5
Female	28	31.5

*Nationality*		
Non‐Saudi	8	9.0
Saudi	81	91.0

*Medical condition*		
No	78	87.6
Yes	11	12.4

*Facility*		
KAUH	41	46.1
KKUH	48	53.9

*Presentation*		
Unwitnessed	29	32.6
Witnessed	60	67.4

*Circumstances*		
Accidental	86	96.6
Intentional	3	3.4
Asymptomatic	27	30.3
Symptomatic	62	69.7

*Time to ER admission (hr)*		
Median (IQR)	120 (60, 360)	

The most frequently reported clinical presentation was nausea/vomiting (38.2%) followed by drooling (20.2%) and abdominal pain (12.4%). Most patients (89.9%) were diagnosed through history and X‐ray. As regards the type of FB, it was coins among 58.4% and a battery button among 11.2%. Further, 48.3% of patients had the FB located in their esophagus, and 29.2% had it located in the stomach. Concerning interventions, over half of patients (53.9%) were only observed, while 43.8% were subjected to endoscopy. Only 4.5% of patients experienced complications, such as ulcer, laceration, and fluid collection, among 2.2%, 1.1%, and 1.1%, respectively. The median LOS was 6.47 (IQR 1.21, 18.73) hours, with 47.2% of patients being admitted (Table 2).

**TABLE 2:** Clinical data and outcome of the studied patients.

Item	*N*	%
*Clinical presentation*		
Drooling	18	20.2
Nausea/vomiting	34	38.2
Abdominal pain	11	12.4
Dysphagia	8	9.0
Throat pain	5	5.6
Odynophagia	4	4.5
Other	14	15.7

*Diagnosis*		
History	3	3.4
History and endoscopy	1	1.1
History and X‐ray	80	89.9
History, X‐ray, and endoscopy	3	3.4
History and CT	1	1.1
CT	1	1.1

*Type of FB*		
Coin	52	58.4
Battery button	10	11.2
Other	27	30.3

*Site*		
Colon	8	9.0
Distal jejunal	1	1.1
Esophagus	43	48.3
Larynx	1	1.1
Rectum	2	2.2
Small intestine	8	9.0
Stomach	26	29.2

*Intervention*		
Direct laryngoscopy	1	1.1
Endoscopy	39	43.8
Observation	48	53.9
Surgical	1	1.1

*Complication*		
No	85	95.5
Yes	4	4.5
Ulcer	2	2.2
Laceration	1	1.1
Fluid collection	1	1.1

*LOS (hr)*		
Median (IQR)	6.47 (1.21, 18.73)	

*Admission*		
No	47	52.8
Yes	42	47.2

Abbreviation: LOS = length of stay.

In univariate logistic regression analysis, female patients showed significantly higher odds of being admitted than males (OR = 2.78, 95% CI: 1.1 to 7.02, *P* = 0.031). Also, symptomatic patients showed significantly higher odds of being admitted than asymptomatic ones (OR = 6.51, 95% CI: 2.18 to 19.48, *p* = 0.001). As for clinical presentation, patients presenting with nausea/vomiting and drooling showed significantly higher odds of being admitted than those with no such conditions, respectively, with OR (95% CI) of 7.92 (2.96–21.17, *p* < 0.001) and 5.38 (1.6–18, *p* = 0.006). Additionally, patients who were only observed showed significantly lower odds of being admitted than those subjected to endoscopy (OR = 0.01, 95% CI: 0.002 to 0.04, *p* < 0.001).

In the multiple regression model, each 1‐year increase in age significantly resulted in increasing the odds of being admitted by 1.12 (95%CI: 1.02 to 1.24, *p* = 0.019). Also, intervention was significantly associated with admission as patients who were only observed showed significantly lower odds of being admitted than those subjected to endoscopy (OR = 0.002, 95% CI: 0 to 0.07, *p* = 0.001) (Table 3).

**TABLE 3:** Logistic regression analysis for factors associated with admission.

Item	Univariate analysis			Multivariable analysis		
	Unadjusted OR	95% CI	*p*‐value	Adjusted OR	95% CI	*p*‐value
Age (years)	1.04	0.99 to 1.09	0.106	1.12	1.02 to 1.24	**0.019**

*Sex*						
Male	Ref			Ref		
Female	2.78	1.1 to 7.02	**0.031**	0.27	0.01 to 5.9	0.404

*Medical condition*						
No	Ref			Ref		
Yes	0.6	0.16 to 2.22	0.446	1.11	0.04 to 27.32	0.951

*Presentation*						
Unwitnessed	Ref			Ref		
Witnessed	1.42	0.58 to 3.47	0.446	9.19	0.66 to 127.23	0.098

*Symptoms*						
Asymptomatic	Ref			Ref		
Symptomatic	6.51	2.18 to 19.48	**0.001**	0.7	0.03 to 16.75	0.826
Time to ER admission (hr)	1	1 to 1.0001	0.697	1	1 to 1.0001	0.698

*Clinical presentation*						
Nausea/vomiting						
No	Ref			Ref		
Yes	7.92	2.96 to 21.17	**< 0.001**	2.29	0.26 to 20.22	0.456

*Drooling*						
No	Ref			Ref		
Yes	5.38	1.6 to 18	**0.006**	1.81	0.07 to 45.05	0.717

*Abdominal pain*						
No	Ref			Ref		
Yes	0.21	0.04 to 1.04	0.056	1.35	0.06 to 32.78	0.855

*Dysphagia*						
No	Ref			Ref		
Yes	3.75	0.71 to 19.71	0.118	0.83	0.05 to 14.56	0.901

*Type of FB*						
Coin	Ref			Ref		
Battery button	0.67	0.17 to 2.64	0.564	0.64	0.02 to 21.65	0.802
Other	0.8	0.31 to 2.04	0.639	0.47	0.03 to 6.39	0.569

*Intervention*						
Endoscopy	Ref			Ref		
Observation	0.01	0.002 to 0.04	**< 0.001**	0.002	0 to 0.07	**0.001**
Other (direct laryngoscopy or surgery)	0.08	0.004 to 1.69	0.106	0.05	0 to 4.57	0.194

*Note:* Statistical significance at *p*‐value < 0.05. Statistically significant.

Abbreviations: CI = confidence interval and OR = odds ratio.

In univariate linear regression analysis, each 1‐year increase in age was significantly associated with increasing LOS by 0.35 (95% CI: 0.17 to 0.53, *p* < 0.001), and symptomatic patients had significantly more LOS than asymptomatic ones (coefficient = 8.42, 95% CI: 3.46 to 13.39, *p* = 0.001). As regards clinical presentation, patients with nausea/vomiting, drooling, and dysphagia had significantly more LOS than those with no such conditions, respectively, with coefficient (95%CI) of 8.85 (4.23–13.48, *p* < 0.001), 5.94 (0.03–11.85, *p* = 0.049), and 9.14 (0.88–17.4, *p* = 0.03); however, patients presenting with abdominal pain elicited significantly less LOS than others (coefficient = −8.05, 95% CI: −15.22 to −0.88, *p* = 0.028). Patients who were only observed had significantly less LOS than those who underwent endoscopy (coefficient = −14.91, 95% CI: −18.71 to −11.11, *p* < 0.001).

In multiple regression analysis, each 1‐year increase in age significantly resulted in increasing LOS by 0.36 (95%CI: 0.19 to 0.53, *p* < 0.001). Further, each 1‐h increase in time to ER admission was significantly associated with increasing LOS by 0.0004 (95% CI: 0 to 0.001, *p* = 0.016). Patients who were only observed had significantly less LOS than those who underwent endoscopy (coefficient = −14.59, 95% CI: −19.65 to −9.53, *p* < 0.001) (Table 4).

**TABLE 4:** Linear regression analysis for factors associated with LOS in hours.

Item	Univariate analysis			Multivariable analysis		
	Coefficient	95% CI	*p*‐value	Coefficient	95% CI	*p*‐value
Age (years)	0.35	0.17 to 0.53	**< 0.001**	0.36	0.19 to 0.53	**< 0.001**

*Sex*						
Male	Ref			Ref		
Female	3.38	−1.8 to 8.55	0.198	−2.22	−6.22 to 1.78	0.272

*Medical condition*						
No	Ref			Ref		
Yes	2.36	−5 to 9.72	0.526	1.24	−4.71 to 7.19	0.679

*Presentation*						
Unwitnessed	Ref			Ref		
Witnessed	−0.95	−6.12 to 4.23	0.717	0.06	−4.03 to 4.15	0.977

*Symptoms*						
Asymptomatic	Ref			Ref		
Symptomatic	8.42	3.46 to 13.39	**0.001**	−2.35	−9.05 to 4.35	0.487
Time to ER admission (hr)	0.0004	0 to 0.001	0.073	0.0004	0 to 0.001	**0.016**

*Clinical presentation*						
Nausea/vomiting						
No	Ref			Ref		
Yes	8.85	4.23 to 13.48	**< 0.001**	2.06	−2.74 to 6.86	0.395

*Drooling*						
No	Ref			Ref		
Yes	5.94	0.03 to 11.85	**0.049**	0.52	−5.02 to 6.07	0.851

*Abdominal pain*						
No	Ref			Ref		
Yes	−8.05	−15.22 to −0.88	**0.028**	−0.56	−8.01 to 6.89	0.881

*Throat pain*						
No	Ref			Ref		
Yes	10.1	−0.22 to 20.42	0.055	4.84	−3 to 12.68	0.222

*Dysphagia*						
No	Ref			Ref		
Yes	9.14	0.88 to 17.4	**0.03**	2.69	−4.35 to 9.74	0.449

*Other*						
No	Ref			Ref		
Yes	2.78	−3.86 to 9.42	0.408	3.57	−2.44 to 9.57	0.24

*Type of FB*						
Coin	Ref			Ref		
Battery button	−0.82	−8.72 to 7.09	0.838	1.66	−3.9 to 7.22	0.554
Other	2.5	−2.93 to 7.94	0.362	−0.1	−4.34 to 4.15	0.964

*Intervention*						
Endoscopy	Ref			Ref		
Observation	−14.91	−18.71 to −11.11	**< 0.001**	−14.59	−19.65 to −9.53	**< 0.001**
Other (direct laryngoscopy or surgery)	−9.06	−21.84 to 3.72	0.162	−10.03	−22.39 to 2.34	0.11

*Note:* Statistical significance at *p*‐value < 0.05. Statistically significant.

Abbreviation: CI = confidence interval.

There was no statistically significant relation between the type of ingested FB and age of patients or intervention method as shown in Table 5.

**TABLE 5:** Relation between type of FB and age and intervention.

Item	Type of FB			*p*‐value
	Coin (*n* = 52)	Battery button (*n* = 10)	Other (*n* = 27)	
Age (years)	5 (4, 7)	3.5 (2.75, 6)	8 (3, 25)	0.096

*Intervention*				
Endoscopy	25 (48.1%)	4 (40%)	10 (37%)	0.596
Observation	25 (48.1%)	6 (60%)	17 (63%)	
Other (direct laryngoscopy or surgery)	2 (3.8%)	0 (0%)	0 (0%)	

*Note:* Numerical data are presented as median (IQR), and categorical data are presented as frequency (%). Statistical significance at *p*‐value < 0.05.

Our investigation revealed a statistically significant relation between site of ingested FB and intervention method (*p* < 0.001), as 94.9% of patients subjected to endoscopy, 10.4% of those under observation, and 50% of those subjected to direct laryngoscopy or surgery had the FB located in the esophagus, and 5.1%, 50%, and 0% had the FB located in the stomach (Table 6).

**TABLE 6:** Relation between site of ingested FB and intervention.

Item	Intervention			*p*‐value
	Endoscopy (*n* = 39)	Observation (*n* = 48)	Other (direct laryngoscopy or surgery) (*n* = 2)	
*Site*				
Colon	0 (0%)	8 (16.7%)	0 (0%)	**< 0.001**
Distal jejunal	0 (0%)	1 (2.1%)	0 (0%)	
Esophagus	37 (94.9%)	5 (10.4%)	1 (50%)	
Larynx	0 (0%)	0 (0%)	1 (50%)	
Rectum	0 (0%)	2 (4.2%)	0 (0%)	
Small intestine	0 (0%)	8 (16.7%)	0 (0%)	
Stomach	2 (5.1%)	24 (50%)	0 (0%)	

*Note:* Numerical data are presented as median (IQR), and categorical data are presented as frequency (%). Statistical significance at *p*‐value < 0.05.

## 4. Discussion

Pediatric FBI continues to pose significant diagnostic and management challenges due to its frequency, variable clinical presentations, and potential for serious complications. Our study analyzed 89 cases over a 10‐year period at KSUMC, providing updated insights into local trends following the introduction of coin currency in 2017 [5]. The predominance of male patients, young age distribution, and high incidence of coin ingestion observed in our study are consistent with findings reported in local and international literature [3, 4, 8, 9].

Although FBI can occur at any pediatric age, it is most commonly observed in children between 6 months and 6 years of age, owing to their exploratory behavior and tendency for oral interaction with objects in their environment [16, 17, 19]. The median age in our cohort was 5 years (IQR 3–8 years), which is broadly consistent with regional literature reporting a mean age of 4.4 ± 2.7 years in a comparable Saudi tertiary center cohort [11], though slightly older than the internationally reported peak of 1–3 years [9, 18]. This modest difference may be explained by the predominance of coin ingestion in our cohort, as older children within the toddler‐to‐school‐age range tend to ingest larger denomination coins reflecting their greater physical access to currency [2]. Additionally, as a tertiary referral center, KSUMC may receive a higher proportion of older children with more complex or symptomatic presentations referred from primary care facilities, which could shift the age distribution upward compared with community‐based centers. Furthermore, longer LOS (*β* = +0.36 h per year, 95% CI 0.19–0.53, *p* < 0.001) and admission (OR = 1.12, 95% CI 1.02–1.24, *p* = 0.019) were significantly correlated with age. With each additional year of age, LOS was prolonged by approximately 0.36 h. This positive association may reflect several interacting clinical and behavioral mechanisms. Older children may ingest larger or more complex objects relative to their gastrointestinal caliber, predisposing to symptomatic impaction rather than asymptomatic passage. Additionally, older children may present with more elaborate clinical histories and a broader differential diagnosis, potentially warranting more prolonged observation and investigation. Delayed presentation may contribute to more advanced mucosal injury, as longer time to presentation has been associated with an increased risk of foreign‐body‐related complications. Failure to witness the ingestion has also been identified as an important reason for delayed presentation [11]. We acknowledge that, with a sample of 89 patients, these explanations are speculative; confirmation in a larger cohort is warranted. Our center is a tertiary hospital, which may explain the shorter LOS compared to other studies due to more efficient management and quicker access to specialized treatment. Several previous studies have reported a higher incidence of FB ingestion among males. For instance, a study conducted in Ilorin found a male‐to‐female ratio of 1.6:1 [18], while a study from China reported that 69.1% of FB ingestion cases occurred in males. This male predominance has been attributed to physiological differences, such as the development of mastication, laryngeal protection, and swallowing coordination [19]. Our findings are consistent with these reports, as males accounted for 68.5% of the cases compared to 31.5% among females (68.5% vs. 31.5%). However, a study conducted in Jeddah contradicted these results, reporting no significant association between gender and FB ingestion (*p* = 0.19) [16]. Despite the higher number of male cases in our study, gender was not significantly associated with hospital admission or LOS, suggesting that gender does not influence the severity or clinical course of FB ingestion. Interpretation of the cohort’s demographic and clinical characteristics should also take into account that 34.1% of eligible pediatric records were excluded due to incomplete documentation; the analytic cohort therefore represents patients with complete documentation rather than the full spectrum of pediatric FB presentations at our institution.

The clinical pattern observed in our study showed that gastrointestinal symptoms, particularly nausea and vomiting, were the most commonly reported, followed by drooling, which aligns with previous literature describing these features as typical early presentations [2]. These manifestations may primarily reflect the presence of an ingested or impacted object rather than independently determining disease severity or the need for intervention.

The type of ingested FBs varies across different countries based on dietary practices, cultural characteristics, and sociocultural factors. Currency in the form of coins was the most prevalent category of FB, followed by a diverse assortment of other FBs [2], while fish bones have been reported as a common FB among the Asian population [13]. Our study showed that the most common FB ingested was coin among 58.4% which was consistent with other studies [2–6, 9–12]. Meanwhile, the button battery contributed to only 11.2% of our study, while it was reported by Chalabi et al. to be the most common ingested FB contributing to 27.8% of cases [17].

Stratified analysis by FB type did not reveal a statistically significant relationship between object type and intervention modality in our cohort. However, this null finding must be interpreted with substantial caution. Button batteries, the object type with the highest recognized clinical urgency, comprised only 10 of the 89 cases (11.2%), and this small subgroup limits the power to detect true differences in management patterns. The clinical urgency of button battery ingestion, particularly with esophageal impaction, is well established in the international literature [6] and is not diminished by the null statistical association observed here. The predominance of coin ingestion in our cohort (58.4%) reflects the local epidemiological shift following Saudi Arabia’s 2017 transition to coin currency [5], and continued monitoring of high‐risk object types remains warranted despite their smaller numerical share.

Our research demonstrated that esophageal FBs were the most common site of impaction requiring endoscopic intervention, accounting for 94.9% of all endoscopic procedures. In contrast, gastric FBs were predominantly managed conservatively, with observation alone sufficient in ∼50% of cases. These findings are consistent with those of Altokhais et al., who also identified the esophagus as the most frequent site necessitating intervention in their review of complicated pediatric cases [2]. Similarly, Nadir et al. and Waltzman et al. reported that esophageal impactions were more likely to require active removal due to the anatomical narrowing and associated risk of obstruction and mucosal injury [3, 4]. In line with our findings, Ibrahim et al. reported that many ingested foreign bodies could be managed conservatively, particularly when they were low‐risk and patients remained asymptomatic [11]. This further supports the recommendations by Mubarak et al. for conservative management of asymptomatic gastric button‐battery ingestions in selected cases, while advocating endoscopic removal when symptoms, delayed diagnosis, or other high‐risk circumstances are present [6]. Collectively, these findings across local and international studies reinforce that anatomical location is a critical determinant of both clinical presentation and management strategy, with esophageal impactions warranting urgent endoscopy and gastric locations often allowing for noninvasive observation.

The site of impaction emerged as the principal determinant of hospital resource utilization in pediatric FBI. In our series, the esophagus was the most frequent lodging site in 48.3% of the cases. This finding is consistent with anatomical considerations, as the upper esophagus—the narrowest portion of the pediatric alimentary tract—acts as a natural catch‐point for ingested objects [5]. These findings are consistent with previous studies [2, 4, 5, 8]. However, regional variations exist: Two studies done in Saudi Arabia in 2019 and 2023 found that the predominant site of impaction is the stomach [11, 16], whereas Ref. [20] in Pakistan reported that 60.8% of cases are in the upper esophagus [20]. Such discrepancies likely reflect differences in patient populations, referral patterns, or diagnostic practices.

In our study, complications were identified in 4 out of 89 patients (4.5%). All four complications were classified as disease‐related (i.e., attributable directly to mucosal contact with the ingested FB) rather than procedure‐related. Documented adverse findings included esophageal ulceration in two cases (2.2%), laceration in one case (1.1%), and fluid collection in one case as well (1.1%), all considered minor complications. No procedure‐related adverse events, including postendoscopy perforation, hemorrhage, or anesthetic complications, were recorded in any patient across the cohort. This distinction is clinically significant, as immediate complications of paediatric upper endoscopy have been reported in approximately 2.3% of procedures, with hypoxia (1.5%) and bleeding (0.3%) being the most commonly reported complications [21], and separating them from disease‐related mucosal injury is important for accurate safety reporting in future studies. The observed complication rate is lower than that reported in other cohorts. For example, one study involving 240 pediatric patients with esophageal FBs documented complications in 10% of cases, predominantly esophageal perforation [2]. Another study reported a 9.3% complication rate, including mucosal abrasions, bowel obstruction, and varying degrees of gastrointestinal injury, with coins accounting for nearly half of the injuries [9]. Notably, our series did not include any major complications secondary to coin ingestion. This is in line with prior findings suggesting that serious outcomes are more likely associated with high‐risk objects, such as button batteries [15]. In alignment with international consensus guidelines, endoscopic removal of esophageal button batteries should be performed within 2 hours of presentation to prevent irreversible mucosal injury. The ESPGHAN position paper recommends immediate endoscopic removal of button batteries impacted in the esophagus, preferably within 2 hours, regardless of symptom status, given the risk of rapid electrochemical tissue injury caused by hydroxide ion generation at the battery’s negative pole [6]. The relatively low rate of complications in our cohort may reflect timely endoscopic intervention, effective triage in the emergency department, and a lower prevalence of hazardous FBs. Moreover, the nature of our institution as a tertiary care center likely contributed to these outcomes. Immediate access to specialized pediatric surgical and endoscopic teams, in addition to streamlined referral pathways, may have facilitated earlier diagnosis and management, thereby limiting the extent of injury. These findings underscore the importance of early identification and stratification based on the nature of the ingested object. While the overall risk of complications appears limited in low‐risk ingestions, vigilance remains essential, particularly in cases involving batteries or delayed presentation.

In our study, the median time to emergency department presentation was 120 h (IQR 60–360 h), representing a notably prolonged delay that warrants contextualization. Several factors likely contributed to this pattern. First, 32.6% of ingestion events were unwitnessed, meaning caregivers were unaware of the event until symptoms emerged. Second, a proportion of caregivers may have adopted a wait‐and‐see approach, particularly for asymptomatic children or in anticipation of spontaneous passage without seeking medical attention. This pattern of delayed presentation has been documented in other regional studies from Saudi Arabia and the broader Middle East [10, 11], suggesting it reflects a consistent regional health‐seeking behavior rather than an isolated finding. Despite this, delayed presentation did not significantly increase the odds of intervention or complications in our cohort, contrasting with previous evidence suggesting that delayed presentation or extraction is associated with an increased risk of adverse outcomes [9]. This discrepancy may reflect the predominantly low‐risk object profile in our cohort (i.e., coins accounted for 58.4% of cases) as well as the rapid triage and management upon arrival afforded by a tertiary center setting. Nevertheless, for high‐risk objects, such as button batteries and sharp items, any delay in presentation carries a substantially elevated risk of serious mucosal injury, and public health messaging advocating immediate presentation following witnessed or suspected ingestion of such objects remains an important preventive priority.

Radiological imaging constituted the primary diagnostic approach in this study, with the majority of cases (89.9%) diagnosed through a combination of history and X‐ray. This finding is consistent with previous studies, which also reported plain radiographs of the neck, chest, and abdomen as the most commonly used initial diagnostic tool in cases of suspected FB ingestion, particularly for radiopaque objects, such as coins and button batteries [1, 2, 6, 8, 15]. Endoscopy was utilized both diagnostically and therapeutically in a subset of patients, especially when radiographic findings were inconclusive or when clinical symptoms persisted despite negative imaging. This approach is supported by regional data from Abualenain et al. [8], who reported X‐ray usage in 87% of cases, and noted that CT was reserved for patients in whom the diagnosis could not be confirmed radiographically [8]. Similarly, in our cohort, isolated use of CT or endoscopy alone was rare, with endoscopy more commonly used in conjunction with imaging or guided by persistent clinical suspicion. Importantly, it must be acknowledged that a proportion of ingested FBs, particularly radiolucent objects, cannot be confirmed by any noninvasive imaging modality regardless of technique. Plain radiography detects radiolucent objects poorly, and even CT may fail to identify certain low‐density materials, as has been documented in cases where endoscopy retrieved FBs invisible on both plain film and CT [22]. In such patients, upper gastrointestinal endoscopy serves simultaneously as the definitive diagnostic and therapeutic tool. Clinicians must therefore maintain a high index of suspicion based on clinical history, witnessed ingestion, and persistent symptoms, and recognise that negative imaging does not necessarily exclude an esophageal foreign body, particularly when clinical suspicion remains high [7, 14].

## 5. Limitations

Several limitations should be acknowledged in this study. Being conducted at a single tertiary hospital in Riyadh limits the applicability of the findings to other regions of Saudi Arabia. The geographical location and institutional affiliation of the center may restrict access for patients from outside the immediate catchment area under nonemergency circumstances, potentially skewing the case mix toward more complex or referred presentations. However, as a tertiary emergency‐capable center, KSUMC accepts all acute pediatric cases regardless of affiliation, and high‐acuity presentations from the broader region are therefore represented in the cohort. Of the 135 eligible pediatric patients identified, 46 (34.1%) were excluded due to missing data across key variables including diagnostic approach, FB type, anatomical site, intervention, and presentation characteristics. Whether these excluded patients differed systematically from the included cohort, for example, representing more acute presentations with less thorough documentation, cannot be determined, and this represents a potential source of selection bias. Future prospective studies should implement standardized data collection tools at the point of care to minimize documentation gaps and improve cohort completeness. Mortality and recurrence of ingestion events were not captured in this study; the retrospective single‐institution design precluded postdischarge follow‐up and identification of repeat presentations at other facilities, and these outcomes should therefore be prospectively assessed in future multicenter studies. In addition, emergency‐specific outcomes, including complication timing relative to ingestion, granular emergency department LOS stratified by triage acuity, and pediatric intensive care unit admission, were not consistently captured in the retrospective records and are therefore not reported. Future prospective studies should incorporate these outcomes using standardized emergency medicine data collection instruments to enhance clinical applicability. The classification of ingestion as “intentional” is a further limitation; this designation was based solely on caregiver‐reported history and may reflect caregiver interpretation rather than true patient intent. Future studies should apply validated behavioral assessment tools to distinguish accidental exploratory ingestion from deliberate self‐harm, particularly in older children. Additionally, regression models may be limited by the small sample size relative to the number of covariates; with 89 patients and 42 admission events across 12 predictor variables, there is a potential for overfitting and unstable estimates, as reflected in the wide confidence intervals observed in several multivariable results. Collinearity between symptom variables should also be interpreted with caution. Accordingly, the regression findings should be regarded as exploratory and hypothesis‐generating rather than confirmatory, and validation in larger prospective cohorts is warranted. Moreover, the study focused on a localized patient population rather than a national cohort, so the results may not reflect trends in other healthcare settings. As this was a retrospective study, granular procedural details, including specific endoscopic technique, retrieval instrumentation, anesthetic approach, and the clinical decision pathway leading to each intervention, were not systematically documented in the medical records and therefore could not be extracted or reported. Intervention categories were classified as endoscopic removal, conservative management with expectant observation for spontaneous passage, and other procedures including direct laryngoscopy or surgical endoscopy, based solely on what was documented. Future prospective studies should incorporate standardized procedural documentation to enable more detailed technique‐specific and protocol‐adherence analysis.

## 6. Conclusion

In conclusion, this study offers an updated perspective on pediatric FBI over a decade at a major tertiary center in Saudi Arabia. Over 10 years of reviewing pediatric FBI at our center, coins were identified as the most frequently swallowed items, reflecting the epidemiological shift following Saudi Arabia’s 2017 transition to coin currency. The esophagus was the primary site of lodgment and most often required endoscopic removal, while objects located in the stomach or distal bowel were commonly managed without intervention. Although complications were uncommon, button batteries remained a critical concern because of their potential to cause severe tissue damage. Our analysis also showed that increasing age correlated with longer hospital stays and that the site of impaction influenced the likelihood of intervention, information that may assist in refining triage and treatment strategies.

These findings highlight the need for targeted community interventions, including educating caregivers, promptly identifying high‐risk objects, such as button batteries, and adhering to unified emergency care protocols. Conducting larger, multicenter investigations would provide nationally representative insights and help evaluate whether such preventive strategies effectively reduce complications and improve pediatric outcomes.

## Funding

The author(s) received no financial support for the research, authorship, and/or publication of this article.

## Conflicts of Interest

The authors declare no conflicts of interest.

## Data Availability

Data are available upon request.
